# Emerging role of RNA sensors in tumor microenvironment and immunotherapy

**DOI:** 10.1186/s13045-022-01261-z

**Published:** 2022-04-12

**Authors:** Rui Yang, Sihui Yu, Tianhan Xu, Jiawen Zhang, Sufang Wu

**Affiliations:** 1 Department of Obstetrics and Gynecology, Shanghai General Hospital, Shanghai Jiao Tong University School of Medicine, Shanghai, People’s Republic of China; 2 Reproductive Medicine Center, Department of Obstetrics and Gynecology, Shanghai General Hospital, Shanghai Jiao Tong University School of Medicine, Shanghai, People’s Republic of China

**Keywords:** RNA sensor, Tumor microenvironment, Immunotherapy

## Abstract

RNA sensors detect foreign and endogenous RNAs to protect the host by initiating innate and adaptive immune response. In tumor microenvironment (TME), activation of RNA sensors induces tumor-inhibitory cytotoxic T lymphocyte responses and inhibits the activity of immunosuppressive cells though stimulating type I IFN signaling pathway. These characteristics allow RNA sensors to be prospective targets in tumor immunotherapy. Therefore, a comprehensive understanding of the roles of RNA sensors in TME could provide new insight into the antitumor immunotherapy. Moreover, RNA sensors could be prominent triggering targets to synergize with immunotherapies. In this review, we highlight the diverse mechanisms of RNA sensors in cancer immunity and their emerging contributions in cancer immunotherapy, including monotherapy with RNA sensor agonists, as well as combination with chemotherapy, radiotherapy, immune checkpoint blockade or cancer vaccine.

## Introduction

Activation of pattern recognition receptors (PRRs), a kind of germline-encoded host sensors, produces type I interferon and interleukin-1β (IL-1β) in the innate immune system [1]. The PRRs sense nucleic acids are called nucleic acid sensors, and can be divided into two categories: one is sensors that detect nucleic acids in endosomes, such as Toll-like receptor (TLR) family members, another group is represented by sensors that detect nucleic acids in cytosol, such as retinoic-acid inducible gene-I (RIG-I)-like receptors (RLRs) and cyclic GMP-AMP synthase (cGAS) [2–5]. According to the sensed nucleic acid types, there exist two types of nucleic acid sensors, defined as DNA sensors and RNA sensors [2]. These sensors detect exogenous and endogenous nucleic acids not only responding to cellular stress, damage and destruction of homeostasis, but also mediating innate immunity and antitumor immunity.

TLRs and RLRs are two classical receptor families that act as RNA sensors. TLRs family members localize to cell surface and endosomes, among which TLR3, TLR7 and TLR8 function as RNA sensors. TLR3 recognizes double-stranded RNA (dsRNA) of various viral genome or replication intermediates, and boosts host immune response to viral infection [6, 7], whereas TLR7 and TLR8 recognize guanosine and uridine-containing single-stranded RNA (ssRNA) motifs in viral RNA [8]. When combined with RNA, the RNA sensors of TLRs switch through conformational changes, recruit adaptor protein TRIF for TLR3 and MyD88 for TLR7/TLR8, causing IRF3/7 phosphorylation and IFN-β secretion [9]. RLRs predominately localize to the cytoplasm and consist of three members: RIG-I, melanoma differentiation-associated protein 5 (MDA5), and laboratory of genetics and physiology 2 (LGP2) [10–12]. RIG-I recognizes 5’-triphosphate RNA (3pRNA) in the cytoplasm of virus-infected cells, and initiates antiviral immune response by activating MAVS and TBK1 signals [13–15]. MDA5 recognizes longer dsRNA, while LGP2 facilitates the interaction between MDA5 and dsRNA [5]. After binding to RNA, RIG-I and MDA5 undergo conformational modifications, induce type I IFN and immune related genes through direct activation and recruitment domains CARD-CARD mediated interactions with mitochondrial associated adaptor protein MAV, and activate IRF3 and NF-κB-mediated signal cascades [16–18]. The characteristics of these RNA sensors are listed in Table 1. Although RNA sensors are important for rapid detection of viral RNAs and restrain of viral replication and transmission, they have the risk of potential recognition of self-RNAs, which might generate autoimmune diseases. Therefore, the dynamic regulation of RNA sensors is essential to prevent inappropriate recognition of endogenous RNA [19].

**Table 1:** Summary of RNA sensors

RNA sensors	Ligands	Cell distribution	Subcellular localization	References
TLR3	Virus genome dsRNA	Myeloid cells, antigen-presenting cells, cancer cells	Endosomes	[2, 5, 6]
TLR7	Guanosine ssRNA	Dendritic cells, macrophages, B cells, cancer cells	Endosomes	[5, 7, 37–42]
TLR8	Uridine ssRNA	Monocytes, macrophage, dendritic cells, cancer cells	Endosomes	[5, 7, 45, 51]
RIG-I	5’-triphosphate RNA (3pRNA)	Intestinal epithelial cells, astrocytes, cancer cells	Cytosol, nucleus	[2, 5, 12–14, 56–59]
MDA5	Longer dsRNA	Intestinal epithelial cells, cancer cells	Cytosol	[2, 5, 12, 58, 59]
LGP2	dsRNA	Dendritic cells, cancer cells	Cytosol	[2, 5, 69]
NOD2	Viral genomic ssRNA	Hematopoietic and non-hematopoietic cells	Cytosol	[5, 70, 71]

As RNA sensors can recognize pathogen-associated molecular patterns (PAMPs) and induce protective immunity to protect host from foreign intruders, more and more researchers have paid attention to their roles in cancer immunity. Accumulating evidences have suggested that RNA sensors within human cancer cells can respond to cytosolic RNA to induce type I IFN signals, and trigger antitumor immunity and tumor clearance [20]. Nowadays, RNA sensors have been widely used in cancer immunotherapy attributed to their anti-tumor potentiality. Herein, we summarize the roles of RNA sensors in cancer immunity, especially their expression and interaction with immune cells in tumor microenvironment (TME), and describe the insights and emerging cancer immunotherapy strategies based on RNA sensors.

## RNA sensors contribute to cancer immunity

### Toll-like receptors

**Fig. 1:**
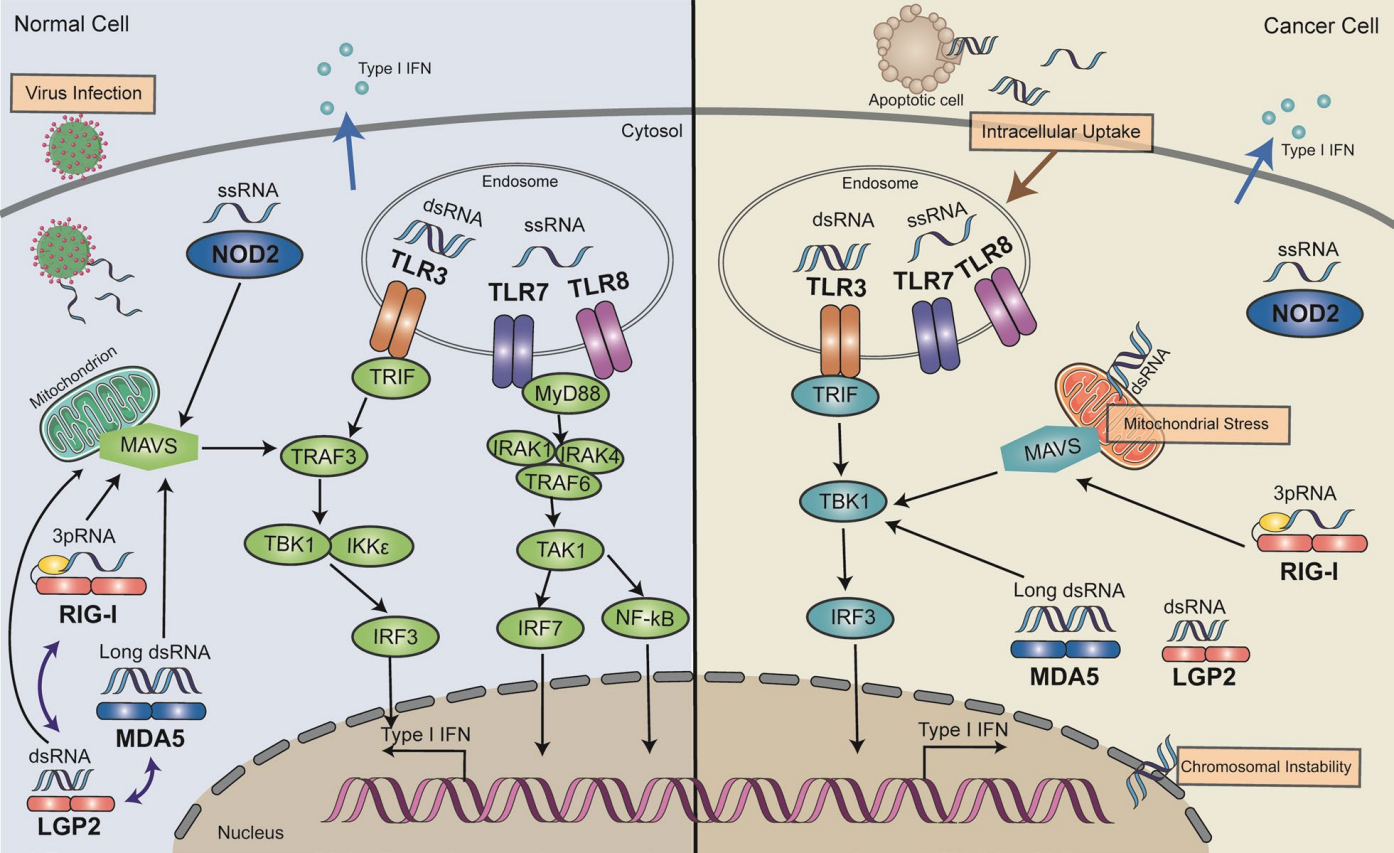
Signaling pathways of RNA sensors in normal and cancer cells. RNA derived from virus infection, intracellular uptake, mitochondrial stress, chromosomal instability can be sensed by RNA sensors. RNA-sensing TLRs, including TLR3, TLR7 and TLR8, predominately localize to the endosome. All RNA-sensing TLRs form homodimers upon activation. TLR3 recruits TRIF to activate the kinases TBK1 and IKKE via activation of TRAF, resulting in phosphorylation and activation of the transcription factor IRF3 to drive type I IFN expression. TLR7 and TLR8 recruit MyD88. MyD88 then recruits the kinases IRAK4 and IRAK1, activates TRAF6 and TAK1, resulting in activation of IRF7 and NF-kB which drives IFN-I expression. RIG-I, LGP2, MDA5 and NOD2 recognize intracellular RNA, bind to the mitochondrial located adaptor protein MAVS, and trigger the activation of TRAF3 to activate TBK1 and drive type I IFN expression. RIG-I senses 3pRNA though MAVS signaling pathway and MAD5 induces IFN responses via TBK1-IRF3 pathway

TLRs are primarily expressed on immune cells and stimulate innate and adaptive immune responses against pathogens by activating various downstream signaling cascades to induce cytokines and chemokines secretion [21]. Besides, TLRs also express on malignant epithelial cells and mediate apoptosis in several tumors [22]. However, TLR RNA sensors have different cellular expression profiles and intracellular signal pathways, raising the possibility that distinct TLRs differentially influence the TME. Similar to immune cells, cancer cells respond to TLR ligands by secreting cytokines and chemokines, which enhance the recruitment and activation of immune cells (Fig. 1).

TLR3 is one of the TLR targets that represents the potential of anti-tumor activity, and its expression is significantly increased in tumor tissues [23]. Studies have shown that the responsive tumors are characterized by up-regulation of STAT1 and TLR3 signaling, down-regulation of IL-10 signaling, and more infiltrating-activated natural killer (NK) cells [24]. TLR3 in TME is mainly expressed in Batf3-positive dendritic cells (DCs) (CD141^+^ DCs in human, CD8a^+^ and CD103^+^ DCs in mouse) and tumor associated macrophages (TAMs) [25–27]. In human malignant tumors, CD141^+^ DCs (cDC1) were essential to induce tumor-inhibitory cytotoxic T lymphocyte (CTL) responses [28–30]. TLR3 stimulation by poly-IC has been found to expand and activate cDC1 by inducing IFN-λ1 production, and recruit cytotoxic effector cells in TME [31, 32]. In non-small-cell lung cancer (NSCLC), the expression of TLR3 on cancer cells contributed to stimulated CD103^+^ lung dendritic cell subset, activated caspase-3 and induced apoptosis [33]. TAMs are the main component of TME, and known to release tumor necrosis factor-α (TNF-α) in response to poly-IC to induce cell death [26, 31, 32]. Vidyarthi et al. [34] reported that TLR3 stimulation reverted macrophages phenotype from M2 type to M1 type and regressed tumor growth via IFN-α/β signaling pathway. In melanoma, TLR3 agonists could induce antitumor immunity by activating NK cells to hinder B16 melanoma lung metastasis [35, 36], whereas the activation of NK cells in lung was mediated by alveolar macrophages and shapes macrophage behavior [37]. Moreover, TLR3 activation decreased the expression of PD-L1, ablated FoxP3 positive CD4^+^ T cells and elicited a distinct CD8^+^ T cell activation phenotype in TME [38]. However, TLR3 activation have also been reported to promote cancer progression. For example, lung cancer microparticles (L-MPs) activated TLR3 and NLRP3 inflammasome, induced macrophages to release IL-1β, and thus promoted lung cancer development [39]. In addition, cancer cells induced chemotactic signaling pathway in endothelial cells by activating TLR3-SLIT2 axis [40].

TLR7 is usually expressed in endosomes of different immune cells, including B lymphocytes, plasmacytoid and conventional dendritic cells (pDCs and cDCs) and macrophages [41]. High expression of TLR7 is reported to be associated with poor prognosis of cancerous patients [42–46]. TLR7 not only played an immunosurveillance role on activation of innate and adaptive immune effectors, but also exhibited a dual regulatory effect on tumor progression [47]. On the one hand, TLR7 activation recruited immunosuppressive cells to facilitate tumor-immune escape. For example, TLR7 stimulation in cancer cells favored tumor progression through increasing the secretion of C–C motif chemokine ligand 2 (CCL2) and granulocyte–macrophage colony stimulating factor (GM-CSF) in TME and eliciting the recruitment of myeloid derived suppressor cells (MDSCs) into the tumor [42]. On the other hand, TLR7 stimulation promoted immune cell infiltration in TME, which functioned as a tumor suppressor. For instance, increased TLR7 expression indicated poor prognosis and was positively correlated with immune cell infiltration (such as T cells, macrophages, neutrophils and DCs) and immune checkpoint expression in stomach adenocarcinoma [46]. Additionally, TLR7 also has the potential to induce CD4^+^ T cells and CD8^+^ T cell infiltration into TME [48].

TLR8 is expressed in the endosomal compartment and is significantly enriched in monocytes, macrophage and DCs [49]. TLR8 signaling could reverse the suppressive functions of tumor-derived CD4^+^ T cells, CD8^+^ T cells and γδ regulatory T (Treg) cells resulting in enhanced anti-tumor immunity [50–55]. Activation of TLR8 in cancer cells is found to prevent the induction of senescence in responder T cells and DCs [56], stimulate glucose uptake and glycolysis in CD4^+^ T cells [57], and induce apoptosis of MDSCs to enhance the anti-tumor effects of adaptive immune response [58]. TLR8 activation also stimulated the release of distinct inflammatory mediators, such as Th1-polarizing cytokines and chemokines, skewed monocytes toward an M1 phenotype and reversed MDSC-mediated suppression of T cell proliferation [59, 60]. Moreover, Safarzadeh et al. [61] reported that TLR7/8 agonist reduced the immunosuppressive activity of patient-derived MDSCs on T cells via promoting MDSCs repolarization into mature myeloid cells.

### RIG-I-like receptors

RLRs are cytosolic PRRs which can sense cytosolic RNA, and have been found to be expressed in several human normal and cancer cells [62–67]. RIG-I signaling activation promotes immune activation in TME, drives transcriptional activation of pro-inflammatory genes involving type I IFNs and pro-inflammatory cytokines and results in immunogenic cell death [68]. Previous studies demonstrated that RIG-I sensitized cancer cells to irradiation treatment by interacting with XRCC4 to compromise virus integration and DNA repair [66]. Activation of MDA5 and RIG-I induced apoptosis in colorectal cancer through mitochondrial pathway [67]. Recently, the role of RLR RNA sensors on antitumor immunity has also been revealed (Fig. 1).

In melanoma, RIG-I signaling triggered surface expression of membrane-bound TNF-related apoptosis-inducing ligand (TRAIL) in naïve NK cells, and induced a TRAIL-dependent cytotoxic NK cell response [69]. In humanized lung cancer model, RIG-I activated by MAPK/IRF1 triggered an interferon and a pro-apoptotic response, resulting in the reduction of exhausted CD8^+^ T cells and tumor shrinkage [70]. Overexpression of RIG-I in hepatocellular carcinoma (HCC) promoted the polarization of M1 macrophages in vitro and increased cancer cell apoptosis in vivo through RIG-I/MAVS/NF-κB pathway [71]. In a hypoxic murine melanoma model, RIG-I was activated and has been found to provoke melanocyte antigen-specific CD8^+^ T cells and NK cells attack, and enhance 3pRNA antitumor efficacy [72]. However, RIG-I also contributed to tumor immune escape. For example, high expression of RIG-I predicted worse clinical outcome in ovarian cancer, and was correlated with immune-regulatory signatures involving checkpoint molecules (PD-L1/PD-1) [73]. In nasopharyngeal carcinoma, Epstein-Barr virus (EBV)-encoded circBART2.2 induced PD-L1 transcription via binding the helicase domain of RIG-I and activating NF-κB and IRF3 cascades, leading to immune escape [74].

MDA5 is another important RNA sensor of RLR family which recognizes longer dsRNA in the cytosol [13]. Activation of MDA5 generated type-I IFN in various DC subsets, and enhanced cytotoxic T cell expansion [75]. Recently, Brown et al. [76] reported that MDA5 could also orchestrate TBK1-IRF3 signaling and sustain type-I/III IFN release, helping Th1 differentiated antitumor T cell phenotypes in TME.

LGP2 poses dual regulating effect of RNA sensing. In neuroblastoma cells, ectopic expression of LGP2 significantly promoted poly-IC‐induced cell death and was associated with downregulation of RIG‐I, MDA5 and MAVS [77]. In breast cancer patients who received radiotherapy, DCs in TME were correlated with LGP2 expression and linked to the clinical outcome. The absence of LGP2 in DCs inhibited the production of type-I IFN and the priming capacity of DCs, and impaired the function of tumor infiltrating CD8^+^ T cells [78].

### Other RNA sensors

Besides, some emerging RNA sensors have been revealed and defined, including NOD-like receptors (NLRs), heterogeneous nuclear ribonucleoproteins (hnRNPs), DEAD-box or DEAH-box RNA helicases and ZBP1 [5, 10]. These RNA sensors are found to sense RNA and interact with TLRs and RLRs in innate immunity. However, few studies have reported their relationship with antitumor immune response.

NOD2, a member of NLRs family, has been demonstrated to function as an RNA sensor by recognizing viral genomic ssRNA and regulate IRF3-dependent antiviral immunity responses via MAVS pathway in both hematopoietic and non-hematopoietic cells [79]. Dysregulation of NOD2 has also been reported in tumorigenesis. In lung adenocarcinoma, cancer cells induced decreased NOD2 expression, resulting in the phenotypic polarization of macrophages through NF-κB signalling pathway [80]. Recently, cGAS-like receptors (cGLRs) are shown to recognize distinct molecular patterns and catalyze synthesis of different nucleotide second messenger signals. In drosophila, cGLRs could sense dsRNA and induce an enhanced antiviral response by synthesizing 3′2′-cGAMP [81]. A study found that chicken Asp-Glu-Ala-Asp (DEAD)-box helicase 1 (DDX1) was an RNA sensor in antiviral innate immunity and mediated IRF7-IFN-β signaling pathway [82]. Poly (ADP-ribose) polymerase 9 (PARP9), a member of PARP family, served as a non-canonical sensor for dsRNA in human or mouse DCs and macrophages to produce type I IFN via activation of the phosphoinositide 3-kinase (PI3K) and AKT3 pathway [83].

**Fig. 2:**
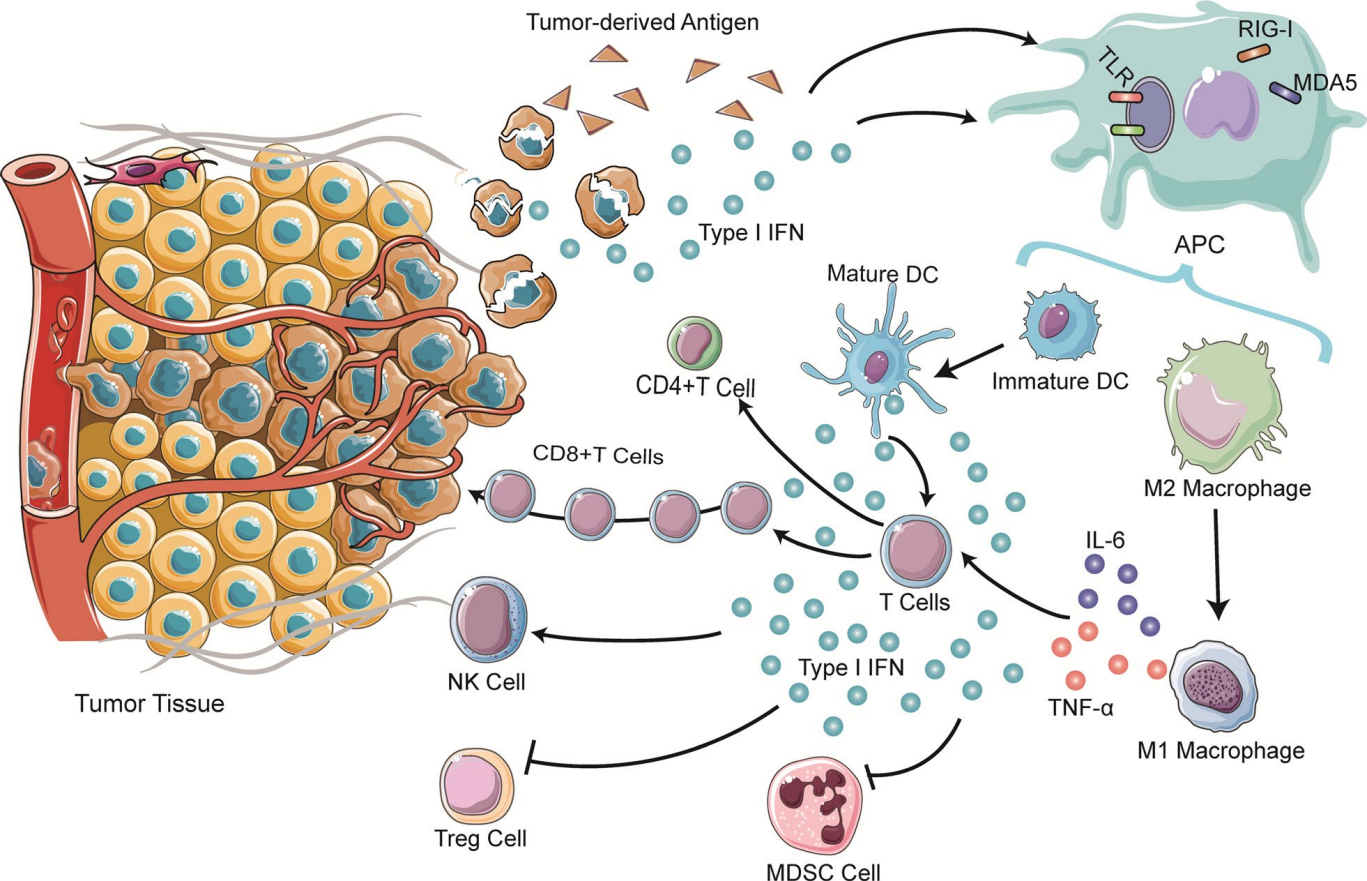
Model for RNA Sensing in the context of anti-tumor immunity. RNA sensors can induce anti-tumor efficacy through multiple mechanisms. Tumor-derived Type I IFN and antigen which contain RNA activates antigen-presenting cells (APC), which mainly including DCs and macrophages. RNA sensors in APCs sense RNA and promote DCs mature and macrophage M1 polarization. Then these cells trigger production of type I IFN and other proinflammatory factors to promote an antitumor immune microenvironment by activating T cells, NK cells and inhibiting Treg and MDSC cells

To sum up, RNA sensors have been found to contribute to cancer immunity across multiple cancer types (Table 2). The activation of RNA sensors in TME plays positive and negative regulatory roles, and interacts with immune cells, making them attractive targets in cancer immunotherapy (Fig. 2).

**Table 2:** The roles of RNA sensors in cancer immunity

RNA sensors	Cancers	Effect on immune microenvironment	Outcome	References
TLR3	NSCLC	DC, NK, CTL activation, macrophage M1 polarization	Suppression	[23, 30, 34]
	Breast cancer	DC, CTL activation IL-12, IFN-γ production	Suppression	[28]
	Lung cancer	IL-1β release	Progression	[36]
	Melanoma	DC, NK cells activation PD-L1 decrease	Suppression	[29, 32, 33, 35]
	Colon cancer	Macrophage M1 polarization	Suppression	[31]
	Breast and lung cancer	Chemotactic signalling pathway	Progression	[40]
TLR7	Melanoma	TAM decrease	Suppression	[35]
	NSCLC	MDSCs recruitment	Progression, metastasis	[38]
	STAD	T cells, macrophages, NK and DC infiltration	Suppression	[42]
TLR8	Prostate cancer	CD8^+^ Treg cells suppression	Suppression	[46]
	Breast cancer	Gammadelta T cells, Treg, MDSCs suppression	Suppression	[48, 49, 55]
	Melanoma	Treg cells suppression	Suppression	[50]
	Solid tumor cells	Tumor-induced T cell and DC senescence prevention, MDSCs apoptosis	Suppression	[51, 52]
	Head and neck cancer	MDSCs suppression, M1 monocyte and CD8^+^ T cells infiltration	Suppression	[54]
RIG-I	Melanoma	CD8^+^ T cells, NK cells response	Suppression	[60, 62]
	Lung cancer	Reduction of exhausted CD8^+^ T cells	Suppression	[70]
	Hepatocellular carcinoma	Macrophages M1 polarization	Suppression	[61]
	Ovarian cancer	IFN production, PD-L1 over-expression	Progression	[63]
	Nasopharyngeal carcinoma	PD-L1 over-expression	Progression	[64]
MDA5	Melanoma	IFN and IL15 production, CTL expansion	Suppression	[65]
	Solid tumor cells	Th1 T cells differentiation, IFN response	Suppression	[66]
LGP2	Breast cancer	DC activation, IFN-1 production, CD8^+^ T cells infiltration	Suppression	[67]
NOD2	Lung adenocarcinoma	Macrophages M2 polarization	Progression	[71]

## RNA sensors in cancer immunotherapy

**Fig. 3:**
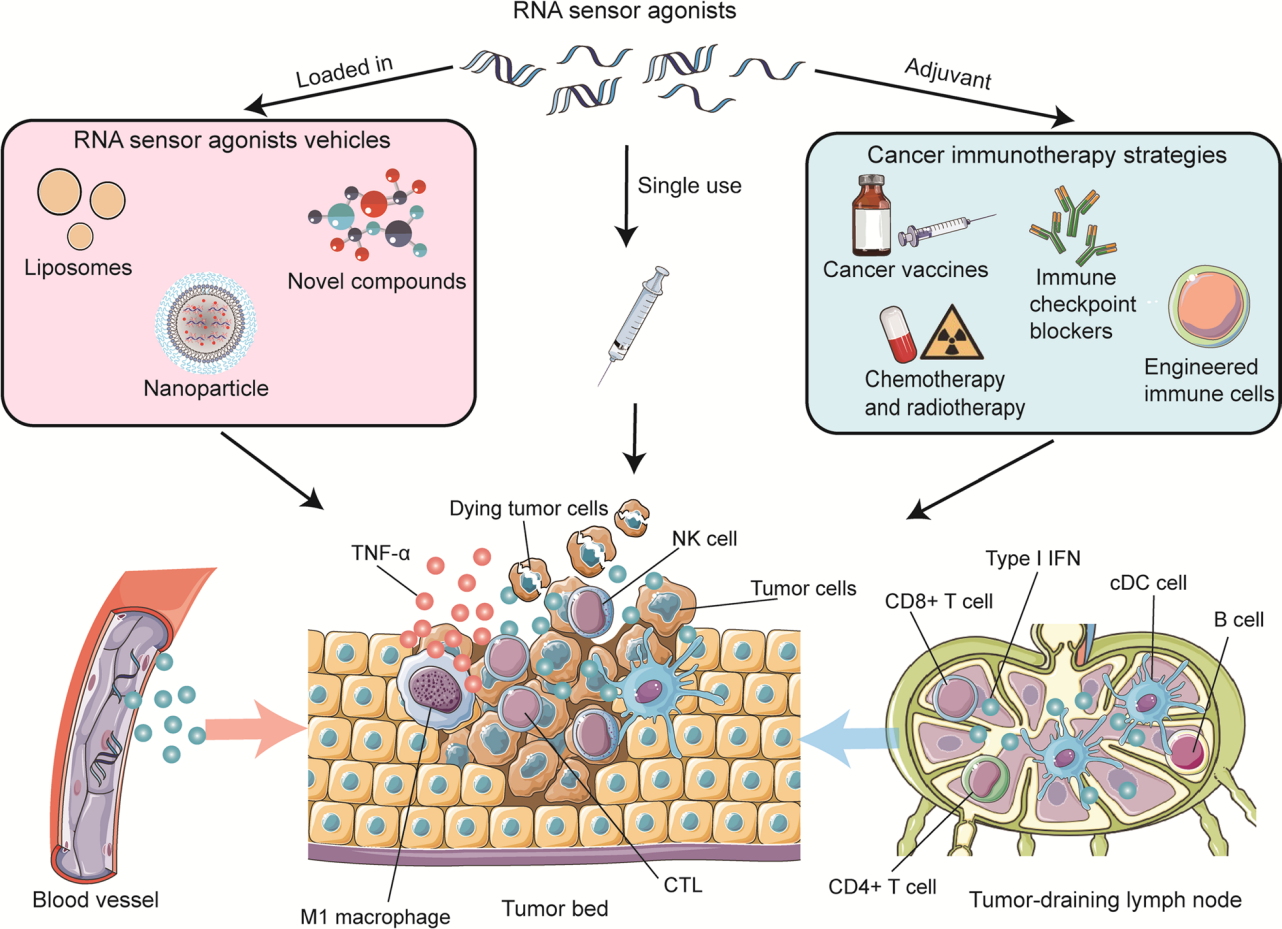
Contribution of RNA sensors in cancer immunotherapy. RNA sensor agonists singly use benefit to cancer immunotherapy by boosting anti-cancer immune response. When loaded into some vehicles such as liposomes, nanoparticle and some novel compounds, they get more effective drug concentration in tumor bed. RNA sensor agonists can also act as adjuvant for other cancer immunotherapy strategies including cancer vaccines, immune cells engineering, immune checkpoint therapy and chemoradiotherapy to induce antitumor immune microenvironment, ameliorate the treatment efficacy and reduce the side effects

As most RNA sensors have function on anti-tumor immunity based on their induction of IFN signaling [84], many drugs targeting RNA sensors are under clinical evaluation for cancer treatment (data from https://clinicaltrials.gov/) (Table 3). Not only local delivery of the single use of RNA sensor agonists, but also the combination with other cancer immunotherapy strategies give great contribution to cancer immunotherapy (Fig. 3).

**Table 3:** Registered clinical trials targeting RNA sensors for cancers (https://clinicaltrials.gov/)

Target	Drug name	Cancer	Combination agent	Phase	Start date/last update	Status	NCT number
TLR3 MDA5	Poly-ICLC (Hiltonol®)	Melanoma	NY-ESO-1, Montanide	I/II	March 2010/February 2018	Completed	NCT01079741
		Melanoma	NA	I	February 2013/October 2015	Terminated	NCT01783431
		B cell and T cell lymphoma	NA	I	April 2009/July 2011	Terminated	NCT00880867
		Low-grade B-cell lymphoma	rhuFlt3L/CDX-301	I/II	November 2013/November 2020	Recruiting	NCT01976585
		Non-Hodgkin's lymphoma, Metastatic breast cancer HNSCC	Pembrolizumab, Flt3L, Radiation	I/II	December 2018/October 2021	Recruiting	NCT03789097
		Solid tumor	Durvalumab Tremelimumab	I/II	December 2015/March 2022	Completed	NCT02643303
		Solid tumor	NA	II	April 2015/December 2020	Completed	NCT02423863
		Solid tumor	NA	II	November 2013/January 2018	Terminated	NCT01984892
		Solid tumor	aPD-1 or aPD-L1	I/II	October 2018/February 2021	Terminated	NCT03721679
		Brain tumors	NA	II	August 2010/September 2021	Completed	NCT01188096
		Brain tumors	Peptide vaccine	I	April 2009/December 2015	Completed	NCT00874861
		Brain tumors	IMA950, Varlilumab	I	October 2016/July 2021	Active, not recruiting	NCT02924038
		CNS tumor	IMA 950	I/II	August 2013/April 2016	Completed	NCT01920191
		Primary ovarian cancer Fallopian tube cancer Primary peritoneal cancer	OC-L, Montanide	I	May 2015/April 2020	Terminated	NCT02452775
		Ovarian cancer	Oregovomab	I	May 2017/December 2020	Terminated	NCT03162562
		Prostate cancer	NA	I	August 2017/April 2021	Recruiting	NCT03262103
		Glioma Glioblastoma	DC vaccination Resiquimod	II	September 2010/February 2022	Active, not recruiting	NCT01204684
		Glioblastoma	IMA950, Pembrolizumab	I/II	September 2018/December 2020	Recruiting	NCT03665545
		Low-grade glioma	NA	II	September 2020/February 2022	Recruiting	NCT04544007
		Solid tumor	Pembrolizumab	I/II	July 2016/July 2021	Recruiting	NCT02834052
TLR3 MDA5	BO-112	Melanoma	Pembrolizumab	II	September 2020/February 2022	Active, not recruiting	NCT04570332
TLR3	Rintatolimod	Recurrent ovarian cancer	Pembrolizumab Cisplatin	I/II	November 2018/March 2022	Recruiting	NCT03734692
	Ampligen (rintatolimod)	Ovarian cancer Fallopian tube cancer Primary peritoneal cancer	OC-L/Montanide ISA 51 VG, Prevnar	I/II	March 2011/November 2021	Terminated	NCT01312389
		Breast cancer	Celecoxib, Cyclophosphamide, Doxorubicin, Paclitaxel	I	September 2019/September 2021	Recruiting	NCT04081389
TLR7	Imiquimod	Cervical squamous cell carcinoma	Topical Fluorouracil	I	June 2017/February 2022	Active, not recruiting	NCT03196180
		Breast cancer	NA	II	May 2009/December 2015	Completed	NCT00899574
		Breast cancer	Radiation Cyclophosphamide	I/II	August 2011/November 2021	Completed	NCT01421017
		Solid tumor	Echopulse PD-1	I	October 2019/August 2021	Recruiting	NCT04116320
	RO7119929	Liver cancer	Tocilizumab	I	April 2020/March 2022	Recruiting	NCT04338685
	SHR2150	Solid tumor	Anti-Cancer Agent	I/II	October 2020/October 2020	Recruiting	NCT04588324
	852A	Breast cancer Ovarian cancer Endometrial cancer Cervical cancer	NA	II	April 2006/August 2019	Completed	NCT00319748
	LHC165	Solid tumor	PDR001	I	October 2017/December 2021	Active, not recruiting	NCT03301896
	BNT411	Solid tumor	Atezolizumab Carboplatin Etoposide	I/II	September 2019/July 2021	Recruiting	NCT04101357
	TQ-A3334	NSCLC	Anlotinib	I/II	February 2020/July 2020	Recruiting	NCT04273815
TLR7/8	Resiquimod	Solid tumor	Pembrolizumab	I/II	March 2021/March 2022	Recruiting	NCT04799054
		Tumors	NY-ESO-1	I	January 2009/January 2015	Completed	NCT00821652
	MEDI9197	Solid tumor	Durvalumab	I	September 2015/December 2018	Terminated	NCT02556463
	NKTR-262	Solid tumor	Bempegaldesleukin Nivolumab	I/II	February 2018/March 2022	Active, not recruiting	NCT03435640
	BDB018	Solid tumor	Pembrolizumab	I	April 2021/August 2021	Recruiting	NCT04840394
	NKTR-262	Solid tumor	Bempegaldesleukin Nivolumab	I/II	February 2018/March 2022	Active, not recruiting	NCT03435640
	BDC-1001	HER2 positive solid tumors	Nivolumab	I/II	February 2020/January 2022	Recruiting	NCT04278144
TLR8	VTX-2337 (Motolimod)	Solid tumor	Cyclophosphamide Pegfilgrastim	I	January 2016/September 2018	Terminated	NCT02650635
		Ovarian cancer	Pegylated Liposomal Doxorubicin Hydrochloride Paclitaxel	I	February 2011/December 2014	Completed	NCT01294293
		Low-grade B cell lymphoma	Radiotherapy	I/II	February 2011/September 2019	Terminated	NCT01289210
		SCCHN	Carboplatin Cisplatin 5-fluorouracil	II	April 2013/October 2019	Completed	NCT01836029
		SCC	Nivolumab	I	April 2019/February 2022	Completed	NCT03906526
		Ovarian cancer	Durvalumab, PLD	I/II	May 2015/September 2021	Completed	NCT02431559
		Epithelial ovarian cancer Fallopian yube cancer Primary peritoneal cancer	PLD	II	August 2012/September 2019	Completed	NCT01666444
	SBT6050	HER2 positive solid tumors	Trastuzumab Deruxtecan, Tucatinib, Trastuzumab, Capecitabine	I/II	October 2021/March 2022	Recruiting	NCT05091528
		HER2 positive solid tumors	Pembrolizumab Cemiplimab	I	July 2020/March 2022	Recruiting	NCT04460456
TLR7/8, RIG-I	CV8102	Solid tumors	Anti-PD-1 therapy	I	September 2017/November 2021	Active, not recruiting	NCT03291002
RIG-I	MK-4621	Advanced solid tumors	Pembrolizumab	I	November 2018/February 2022	Terminated	NCT03739138

### RNA sensor agonists single use contributes to cancer immunotherapy

#### Classical RNA sensor agonists

TLR agonists have received considerable attention as promising targets for cancer immunotherapy owing to their ability to convert immunosuppressive TME towards a T cell inflamed phenotype. Poly-IC, an agonist of TLR3, MDA5 and RIG-I, is usually used as an immune adjuvant. It can directly target tumor-resident DCs, mimic natural infection of dsRNA virus, and initiate a strong inflammatory response by recruiting and activating CD8^+^ T cells [85, 86]. Poly-L-lysine stabilized (Poly-ICLC), a synthetic dsRNA and an agonist of TLR3 and MDA5, stabilizes with poly-lysine and carboxymethylcellulose. Poly-ICLC has been evaluated in a large number of clinical trials with the goal to exert anti-tumor immune effect and can be safely applied to patients [86]. Resiquimod (R848), a TLR7/8 agonist, is usually used as an immune adjuvant, which specifically binds to the PAMPs of DCs and promotes the maturation and activation of DCs to improve anti-tumoral immune response [87–89]. In a mice model of head and neck cancer, R848 could recruit immune cells to the tumor and inhibit tumor growth [87]. TLR7/8 agonist imiquimod was proven to activate NK cells to kill tumor cells and induct tumor-specific CD4^+^ T cells, resulting in a strong regression of low MHC-I tumors [90]. Additionally, Wiedemann et al. [91] reported that a small molecule TLR7-agonist SC-1 could activate NK cell responses and restore NK cell-mediated tumor killing effect in vivo. SC-1 also exerted antitumor effect through releasing type I IFN, activating plasmacytoid DCs, polarizing macrophages to M1 phenotype and decreasing MDSC [92]. Motolimod (VTX-2337), a selective small-molecule TLR8 agonist, could alter lymphocyte differentiation and function, stimulate Th1 polarizing cytokines, enhance antibody-dependent cellular cytotoxicity, and promote innate and adaptive antitumor immunity [93–95]. In a clinical trial of head and neck squamous cell carcinoma (HNSCC), motolimod significantly improved the prognosis of patients with HPV-positive oropharyngeal cancer [96].

Two recent studies demonstrated that RIG-I activated by short 5′-triphosphate-modified RNA (ppp-RNA) reduced tumor burden in melanoma and acute myeloid leukemia (AML) model, which was dependent on CD4^+^ T cells, CD8^+^ T cells and the intact MAVS/IFN signaling in the host [97, 98]. Jiang et al. [99] tested the antitumor activity of stem loop RNA 14 (SLR14), a RIG-I agonist, in immunogenic tumor models. They found that tumor growth was delayed and survival was extended in SLR14-treated mice. The numbers of CD8^+^ T lymphocytes, CD11b^+^ cells and NK cells were observed to be increased in the model. Moreover, SLR14 significantly inhibited the growth of nonimmunogenic B16 tumor, and the cured mice developed immunologic memory. Another study in AML showed that RIG-I activation overcame the intrinsic T cell resistance of IFN-sensitive/resistant melanoma and enhanced the clinical effect of immunotherapy [100].

#### Novel RNA sensor agonists

Nowadays, more emerging RNA sensor agonists have been developed and used for safe and effective cancer immunotherapy, including some RNA-based agonists and small-molecule agonists (Table 4). ARNAX, a TLR3-specific RNA agonist, not only established Th1 immunity in TME, but also upregulated genes involved in the recruitment and function of T cells, NK cells and DCs [101–103]. Single-stranded RNA origami (RNA-OG) based on nucleic acid nanotechnology can stimulate a strong immune response via TLR3 signaling pathway. In a mice peritoneal metastatic colon cancer model, RNA-OG was found to induce obvious tumor growth arrest by activating CD8^+^ T cells and NK cells and antagonize the peritoneal immunosuppressive TME. Unlike poly-IC, RNA-OG administration did not significantly produce high level of type-I IFN in blood, nor did it cause apparent toxicity in the animal model, which make it a potential safe and effective RNA sensor agonist for cancer immunotherapy [104]. In a melanoma model, Liu et al. [105] reported that a ssRNA-Pim-3-small hairpin RNA (shRNA) dual-function vector could activate TLR7 by ssRNA fragments to stimulate antitumor immune response, such as activation of CD8^+^ T cells and NK cells and reduction of intratumoral Treg and MDSCs. In addition, several noncoding RNAs and even recombinant virus have been identified as RNA senor inducers. MiR-574-5p derived from small extracellular vesicles activated TLR7/8, thereby decreased PGE2-levels in lung cancer [106]. CircNDUFB2 is reported to be recognized by RIG-I to activate RIG-I-MAVS signaling cascades and recruit immune cells into the TME in NSCLC [107]. PVSRIPO, a neuro-attenuated recombinant poliovirus, shows strong cytotoxicity in infected tumor cells expressing poliovirus receptor CD155. In malignant glioma, PVSRIPO could induce IFN response and elicit antitumor T cell immunity through MDA5-TBK1-IRF3 signaling [76, 108]. As for small-molecule agonists, 17e (CU-CPT17e), a newly discovered small molecule capable of activating TLRs 3, 8 and 9 simultaneously, could induce THP-1 cells to produce various cytokines, such as IL-6, IL-8 and TNF-α, and inhibit the proliferation of HeLa cancer cells [109]. 1V270, a small molecule TLR7-specific ligand conjugated to a phospholipid moiety, is another RNA sensor agonist. It has been shown to induce tumor-specific adaptive immune responses and suppress primary tumor growth in HNSCC and melanoma mice models [110, 111]. Additionally, local 1V270 treatment could activate TAM and convert an immune-suppressive TME to a tumoricidal environment without inducing systemic cytokine [111]. The 1V270 therapy also inhibited tumor colonization in an NK cell dependent manner, which exhibited a suppression role of lung metastasis by inducing tumor-specific adaptive immune responses [112]. MEDI9197 (3M-052), a novel designed lipophilic TLR7/8 agonist, is found to regulate the enrichment and activation of CD8^+^ T cells and NK cells, polarization of Th1 cells and inhibition of tumor growth in multiple syngeneic models [113].

**Table 4:** Novel developed RNA sensor agonists in cancer immunity

Agonists name	Chemical properties	Target	Cancer	Function	References
ARNAX	dsRNA	TLR3	Lymphoma	Involves in the recruitment and function of T cells, NK cells and DCs, overcame anti-PD-1 resistance	[101–103]
RNA-OG	ssRNA	TLR3	Mice peritoneal metastatic colon cancer model	Activates CD8^+^ T and NK cells, antagonizes the peritoneal immunosuppressive TME, produces type-I IFN	[104]
ssRNA-Pim-3-shRNA	ssRNA	TLR7	Melanoma	Activates CD8^+^ T cells and HK cells and reducts intratumoral Treg and MDSCs	[105]
CircNDUFB2	Noncoding RNA	RIG-I	NSCLC	Activates RIG-I-MAVS signaling cascades and recruits immune cells into the TME	[107]
CircBART2.2	Noncoding RNA	RIG-I	Nasopharyngeal carcinoma	Induces PD-L1 transcription, activates NF-κB and IRF3 cascades	[74]
ppp-RNA	RNA	RIG-I	Melanoma and acute myeloid leukemia	Activates CD4^+^ and CD8^+^ T cells, induced PD-L1 expression	[97, 98]
PVSRIPO	Recombinant poliovirus	MDA5	Glioma	Induces IFN response and elicits antitumor T cell immunity	[76, 108]
17e (CU-CPT17e)	Small molecule	TLR3, 8 and 9	HeLa cancer cells	Produces various cytokines	[109]
1V270	Small molecule	TLR7	HNSCC and melanoma mice models	Induces tumor-specific adaptive immune responses	[110, 111]
MEDI9197 (3M-052)	Small molecule	TLR7/8	Melanoma	Regulates the enrichment and activation of CD8^+^ T cells and NK cells, polarization of Th1 cells	[113]

### RNA sensor agonist delivery vehicles ameliorate cancer immunotherapy efficacy

As it is difficult to achieve effective drug concentration in tumors for systemic administration of agonists, some delivery vehicles such as liposomes, nanoparticle and novel compounds for delivering RNA sensor agonists to the target tumor region have been developed.

#### Liposomes

R848 delivered by complement C3-targeted liposomes triggered various signal cascades to increase the expression of cytokines and factors (such as TNF-α, IL-1β, IL-6 and IL-12), leading to the delay of tumor growth in 4T1 tumor-bearing mice [114]. Zhang et al. [115] developed an intravenously-injectable formulation with R848 by using thermosensitive liposomes (TSLs) as a delivery vehicle (R848-TSLs). Combined with local hyperthermia and αPD-1, systemic administration of R848-TSLs could significantly inhibit tumor growth. Local injection of R848-TSLs combined with αPD-1 also showed superior anti-tumor efficacy. They observed that complete regression of neu deletion (NDL) tumors in both treated and abscopal sites was achieved in 8 of 11 tumor bearing mice with enhanced infiltration and accumulation of CD8^+^ T cells in tumors. In another recent study, Wan et al. [116] conjugated the small molecule TLR7 agonist 1V209 with cholesterol (1V209-Cho) and prepared into liposomes (1V209-Cho-Lip). Compared with 1V209, 1V209-Cho-Lip exerted less toxic effect and enhanced transport capacity of lymph nodes (LN). Subsequent in vivo experiments showed that 1V209-Cho-Lip treatment could inhibit the progression of Pan02 pancreatic ductal cancer, 4T1 breast cancer and CT26 colorectal cancer models by eliciting CD8^+^ T cell responses and inducing effective DC activation. In addition, cholesterol conjugation with 1V209 also induced tumor-specific memory immunity to reduce tumor relapse and metastasis. Cationic liposomes loaded with tumor-specific synthetic long peptides (SLPs) and TLR3 ligands as adjuvants could also induce cytotoxicity against target cells in vivo by strongly activating functional antigen-specific CD4^+^ T cells and CD8^+^ T cells [117].

#### Nanoparticle

Nanoparticle is a class of microscopic particle and has been found to possess anti-tumor therapeutic potential by inducing pro-inflammatory TME. It was also used as a vector to deliver cancer vaccines. When synergized with the poly-IC, nanoparticles such as Ferumoxytol and BO-112, were shown to exert anti-tumor therapeutic potential by inducing macrophage activation and enhancing tumor antigen-specific CTLs in TME [118, 119]. In a mouse model, combination of CHP-NY-ESO-1, a nanoparticle complex of cholesteryl pullulan (CHP) and NY-ESO-1 antigen protein, with anti-PD-1 antibody suppressed the growth of NY-ESO-1-expressing tumors. Further phase 1 clinical trial reported that CHP-NY-ESO-1 could induce higher antibody responses in patients with advanced or recurrent esophageal cancer when combined with poly-ICLC [120]. Nanoparticle that delivers TLR7 ligand to tumor-draining lymph nodes can induce a local cytotoxic T cell response [121], leading to the proliferation of tumor antigen-specific CD8^+^ T cells and potent activation of DCs in the sentinel lymph nodes [89, 122]. When co-delivers TLR‑7 agonists with anti-CD47 antibodies, nanoparticles can induce systematic immune responses and superb antitumor efficacy [123]. R848-loaded nanoparticles were proven to effectively deliver drugs to TAMs, induce the shift of macrophages from M2 to M1 phenotype, activate DCs and increase cytotoxic T cells [124, 125]. Moreover, a self-assembling vehicle-free multi-component antitumor nanovaccine (SVMAV) loaded with R848 and STAT3 inhibitors could effectively migrate into lymph nodes, promote CD8^+^ T cell response and DC function not only in primary melanoma, but also in lung metastasis. It is worth noting that neoantigen-specific SVMAV showed stronger antitumor activity than aPD-1 in an orthotopic HCC model [126]. In addition, a semiconducting polymer nanoadjuvant (SPNIIR) can effectively generate heat not only to induce immunogenic cell death and ablate tumors, but also release TLR agonists, which promotes the maturation of DCs and enhances anti-tumor immune response [127]. The application of a nanoparticles/bacteria complex (Ec-PR848) composed of *Escherichia coli*, DOX-loaded PLGA and R848 was shown to polarize macrophages from M2 to M1 phenotype, impair the immunosuppression of TME, and significantly improve the efficacy of immunotherapy [128]. Delivery of 5’-triphosphate RNA together with endosomolytic nanoparticles could induce immunogenic cell death, trigger the expression of type I IFN and proinflammatory cytokines, and increase the infiltration of CD8^+^ T cells through activating RIG-I pathway in CT26 tumor model [129]. Nanoparticle delivery of RIG-I agonist dsRNA also strongly induced the level of pro-inflammatory Th1 cytokines, further increased the proportion of M1 over M2 macrophages, CD8^+^ T cells over regulatory T cells, and reduced the levels of plasma cells and immunosuppressive B regulatory in pancreatic cancer [130]. These findings provide a promising nanoparticle-based immunotherapy approaches for malignant tumors.

#### Novel compounds

Recent years, some novel compounds for the treatment of malignant tumors have been developed and synthesized. RNA sensor agonists can also be loaded in these compounds as adjuvants to promote their antitumor immune responses. CH-NPs, an ionic complex ovalbumin as a model antigen and poly-IC as the adjuvant, could increase intracellular delivery and maturation of DCs, resulting in the activation of antigen-specific cytotoxic CD8^+^ T cells in vivo [131]. Liu et al. [132] developed a galactose-functionalized zinc protoporphyrin IX (ZnPP) grafted poly(l-lysine)-b-poly(ethylene glycol) polypeptide micelles (ZnPP PM) for TAM-targeted immunopotentiator delivery. ZnPP PM loaded by poly-IC could activate T lymphocytes and NK cells, and effectively repolarize TAM in B16-F10 melanoma tumor model. JOC-x is a conjugatable tumor tight junction opener, when conjugated with poly-IC, it could not only recruit and activate of CD8^+^ T cells by targeting DCs, but also play a tumor killing role by initiating apoptosis in tumor cells [133].

### RNA sensor agonists combined with other cancer immunotherapy strategies

#### Cancer vaccines

Cancer vaccines, produced by tumor-derived antigens (such as microparticles, proteins, peptides and mRNA), can deliver tumor antigens to local tumor region, trigger strong antitumor immune response in situ. Protein or peptide vaccine combined with RNA sensor agonists have been demonstrated to induce activation of antigen-specific CD8^+^ T cells that translates into potent antitumor immunity [134–137]. In a murine melanoma model, tumor antigen vaccination based on anti-CD40 and poly-IC increased the number of CD8^+^ T cells in tumor tissue and delayed tumor growth [138]. In cervical cancer, lung cancer and melanoma models, tumor antigens adjuvant with nanoemulsion (NE) loaded with TLR7/8 agonists showed enhanced infiltration of lymphocytes, polarization of tumor-associated M2 macrophages, strong local and systemic anti-tumor immune response, resulting in inhibited tumor growth and prolonged survival [139, 140]. Additionally, studies have reported that TLR7 agonist imiquimod augmented the immunogenicity of peptide vaccine by activating the strong and durable response of CD4^+^ T cells and CD8^+^ T cells in melanoma [141]. Moreover, tumor cell-derived microparticles (TMPs) by oral vaccination activated NOD2 leading to subsequent antitumor T cell responses, inhibited the tumor growth of CT26 colon cancer and B16 melanoma in mice [142]. Koerner et al. [143] recently reported that biodegradable poly (lactic-co-glycolic acid) (PLGA) particles carrying TLR3/RIG-I ligand Riboxxim could potently activate murine and human DCs and elevate tumor-specific CD8^+^ T cell responses, showing effective anti-cancer effect in preclinical tumor models.

#### Immune cell engineering

The combination of RNA sensor agonists and engineered immune cells provides a new immunotherapeutic strategy for solid tumors. In the immunotherapy of chimeric antigen receptor T cells (CAR-T), Di et al. [144] found that poly-IC significantly promoted the higher lytic activity of CAR-T, enhanced the tumor growth inhibition from CAR-T cells after systemic administration in vivo. Moreover, poly-IC reduced the number of MDSC in peripheral blood and spleen, and weakened the immunosuppressive activity of MDSC on proliferation and cytolytic function of CAR-T cells. In one recent reported study, researchers delivered RN7SL1, an endogenous RNA that activates RIG-I/MDA5 signal, through engineered CAR-T cells to promote the expansion and effector memory differentiation of CAR-T cells. They found that when RN7SL1 was deployed in extracellular vesicles, it could selectively transfer to immune cells to restrict the development of MDSC and reduce TGF-β in myeloid cells. Even when heterogenous CAR antigen tumors lack sufficient neoantigens, CAR-T cells still could co-deploy peptide antigens with RN7SL1 to improve efficacy [145]. In addition, Li et al. [54] found that TLR8 could reverse Treg suppression by selectively inhibiting glucose uptake and glycolysis in Treg cells, and then enhance antitumor immunity in a melanoma adoptive transfer T cell therapy model. Antigen sensitized DCs has found to induce antigen-specific CD8^+^ T cell response in vivo, making them as attractive targets for cancer immunotherapies [28, 29, 146]. When cultured in presence of poly-IC, DCs can more effectively enhance T cell responses [147]. Two previous studies found that the combination of a DC-based vaccination and poly-ICLC was well-tolerated in glioblastoma and glioma [148, 149]. Another clinical trial of pancreatic cancer demonstrated that the combination of peptide pulsed DCs and poly-ICLC was safe and could induce a measurable tumor specific T cell population [150]. Additionally, reovirus-activated NK cells combined with cetuximab could synergistically enhance their antitumor cytotoxicity in colorectal cancer, which was dependent on TLR3 and its downstream signals [151].

#### Immune checkpoint therapy

Application of monoclonal antibodies targeting immune checkpoints, such as programmed cell death 1 ligand 1 (PD-L1), integrin-associated protein (CD47) and cytotoxic T lymphocyte associated antigen 4 (CTLA-4) has been found to improve the survival rate of patients with several cancer types [152–154]. However, there are still a large proportion of patients who cannot benefit from immune checkpoint blockade (ICB) therapy, whereas some cancer types even seem to be less sensitive to ICB [155, 156]. In order to achieve effective outcome, some researches have focused on the effect of RNA sensor agonists on ICB [157]. One study found that pretreatment of IFN-γ, TLR3 ligand poly-IC and anti-IL-10 antibody could sensitize tumors to ICB by increasing infiltrating-activated NK cells [24]. In an HNSCC model, Sato-Kaneko et al. [111] demonstrated that the combined treatment of intravenous TLR agonist and PD-1 blockade activated TAMs, induced tumor specific adaptive immune response, and inhibited primary tumor growth and metastasis. In two breast cancer models, PD-1 blockade combined with poly-IC efficiently modulates immune cell profiles, such as increase in CD8^+^ T cells, type-1 conventional DCs, immunogenic M1 macrophages and CD169^+^ macrophages, and reduction in MDSC, plasmacytoid DCs, regulatory T cells and immunotolerant M2 macrophages, which in turn eliminates not only the primary tumor, but also metastasis [158]. TLR3-specific RNA agonist ARNAX could activate tumor-specific CTLs, and overcome anti-PD-1 resistance without cytokinemia when combined with anti-PD-L1 antibody and a tumor-associated antigen [101]. Application of ppp-RNA also induced the expression of PD-L1 on AML cells and established therapeutic sensitivity against PD-1 checkpoint blocking in vivo [97]. Another RIG-I agonist SLR14 could improve the antitumor efficacy of anti-PD1 antibody over monotherapy [100]. In CT26 colon cancer model, nanoparticle conjugated TLR7 agonists could potentiate the efficiency of checkpoint inhibitors targeting PD-1 and CTLA-4, and even promote a long-term specific immunological memory [159]. Another TLR7/8 agonist-based nanovaccine combined with sunitinib and PD-L1 antibody treatment was proven to upregulate activation of CD8^+^ T cells and reduce MDSCs and PD-L1^high^ M2 macrophages in the tumor, leading to enhanced antitumor efficacy in B16F10 and MB49 mice models [160]. Moreover, combining irreversible electroporation (IRE) with intratumoral TLR7 agonist 1V270 and systemic anti-PD-1 blockade not only improved treatment responses, but also eliminated untreated concomitant distant tumors [161]. In addition, anti-CTLA-4 and its combined immunotherapy with anti-PD-1 have also been found to be dependent on the activation of RIG-I, which could induce cross-presentation of tumor-associated antigen by CD103^+^ DCs, caspase-3-mediated tumor cell death and expansion of tumor antigen-specific CD8^+^ T cells [162, 163].

#### Combination immunotherapy

A major challenge of cancer immunotherapy is to develop a durable, effective and tumor-specific immune response without systemic toxicity. The applications of immune adjuvants combined with chemotherapy, radiotherapy and targeting therapy are able to improve clinical efficacy. Emerging studies have revealed that some RNA sensor agonists used as adjuvants can help to boost the antitumor immune microenvironment, so as to ameliorate the treatment efficacy and reduce the side effects.

In formyl peptide receptor 1 (FPR1)-deficient mice, immunotherapy with Poly-IC has been found to restore the deficient chemotherapeutic responses by improving DC and T lymphocyte-mediated anticancer immunity [164]. Wei et al. [165] developed two targeted polymer micelles to deliver immunomodulator imiquimod (R837) and anticancer drug doxorubicin (DOX) to TAMs and tumor cells through intravenous and intratumoral injection. They found that R837 could stimulate the maturation of TAM, induce the anti-tumor immune response in TME. Meanwhile, the release of DOX in the cytoplasm of tumor cells by the chemotherapeutic micelles could also directly induce cancer cell death. Administration of R848 combined with oxaliplatin reversed the function of MDSCs and strengthened antitumor effect of oxaliplatin in colorectal cancer [166], whereas oxaliplatin-based platinum prodrug bearing TLR7 agonist SZU101 enhanced activation of cytotoxic T cells in tumors and contributed to the high anticancer efficiency in breast cancer model [167]. Ringgaard et al. [168] revealed that combining a liposomal oxaliplatin formulation (PCL8-U75) with R848 induced immunological rejection of established tumors by increasing infiltration of Foxp3-T helper cells and cancer antigen-specific cytotoxic T cells. Moreover, the therapeutic effect of radiotherapy in combination with poly-IC was shown to enhance radiation-sensitivity via TNF-α produced by intra-tumor macrophages and CTL induced by TLR3-positive DC [169]. In EBV-positive nasopharyngeal cancer, Poly-ICLC can strengthen cetuximab-based immunotherapy through enhancing NK-mediated IFN-ɣ expression, antibody-dependent cellular cytotoxicity (ADCC) and DC maturation [170]. In syngeneic human CD20 (hCD20)-expressing models of lymphoma, the combination of R848 and obinutuzumab improved the clearance of lymphoma and produced long-term antitumor immune response [171]. In HNSC, encouraging antitumor activity and strong pharmacodynamic response were also observed when TLR8 agonist Motolimod combined with cetuximab [172].

## Conclusion and perspectives

RNA sensors are important for recognizing PAMPs and could help to protect the host from both exogenous and endogenous RNA. Intriguingly, recent studies show that PRR-mediated RNA sensing also occurs in the nucleus and mitochondrion, highlighting an orchestrated multi-compartmental RNA-sensing paradigm [173–176]. The principle has subsequently been used to develop new treatment strategies, making RNA sensors as an important target for cancer immunotherapy. Deep understanding of the mechanism into the activation and regulation of RNA sensors in cancer immunity is necessary for exploring their applications in antitumor immunotherapy. The accepted notion so far is that activation of RNA sensing pathways is able to suppress tumors. Conversely, in some cases, triggering of RNA sensors by their ligands does not result in antitumor immunity but favors tumor progress and immune escape instead [39, 42, 73, 74]. Therefore, more in-depth exploration of their function is needed in future researches.

Immunotherapy has achieved remarkable success in the treatment of malignancy. Recent progresses in the understanding of how RNA sensing signals affect cancer immunity of agonists, antagonists and novel treatment strategies. As we mentioned above, single or combined application of RNA sensor agonists is becoming a potential effective treatment of cancers, and more burgeoning agonists have been found and developed. It is reported that even recombinant viruses and cellular noncoding RNAs are expected to be agonists [74, 76, 106–108]. Although the primary focus lies on RNA sensor agonists in combination with other conventional treatments or as components of cancer vaccines, systematic treatment strategies based on RNA sensors, novel drug delivery methods and innovative combination with other immunotherapies will continue to promote progress in this field.

## Data Availability

Not applicable.

## Abbreviations

PRR: Pattern recognition receptor
IL-1β: Interleukin-1β
TLR: Toll-like receptor
RIG-I: Retinoic-acid inducible gene-I
cGAS: Cyclic GMP-AMP synthase
dsDNA: Double-stranded RNA
ssRNA: Single-stranded RNA
MDA5: Melanoma differentiation-associated protein 5
LGP2: Laboratory of genetics and physiology 2
PAMP: Pathogen-associated molecular pattern
TME: Tumor microenvironment
NK: Natural killer
DC: Dendritic cell
TAM: Tumor associated macrophage
CTL: Cytotoxic T lymphocyte
NSCLC: Non-small-cell lung cancer
TNF-α: Tumor necrosis factor-α
GM-CSF: Granulocyte-macrophage colony stimulating factor
MDSC: Myeloid derived suppressor cell
TRAIL: TNF-related apoptosis-inducing ligand
HCC: Hepatocellular carcinoma
NLR: NOD-like receptor
cGLR: cGAS-like receptor
PARP9: Poly (ADP-ribose) polymerase 9
PI3K: Phosphoinositide 3-kinase
SLR14: Stem loop RNA 14
TSL: Thermosensitive liposome
FPR1: Formyl peptide receptor 1
ADCC: Antibody-dependent cellular cytotoxicity
SLP: Synthetic long peptide
TMP: Tumor cell-derived microparticle
CAR-T: Chimeric antigen receptor T cell
PD-1: Programmed death receptor 1
CD47: Integrin-associated protein
CTLA4: Cytotoxic T lymphocyte associated antigen 4
ICB: Immune checkpoint blockade
